# Correction: Predicting breast cancer treatment response and prognosis using AI-based image classification

**DOI:** 10.3389/fonc.2025.1741682

**Published:** 2025-12-15

**Authors:** Bingyi Wang, Shu Chen, Wei Li

**Affiliations:** 1 Department of Radiation Oncology, Clinical Oncology School of Fujian Medical University, Fujian Cancer Hospital, NHC Key Laboratory of Cancer Metabolism, Fuzhou, China; 2 Department of Gastric Surgery, Clinical Oncology School of Fujian Medical University, Fujian Cancer Hospital, NHC Key Laboratory of Cancer Metabolism, Fuzhou, China; 3 Medical School, Yangzhou University, Yangzhou, China

**Keywords:** breast cancer prognosis, treatment response prediction, latent dynamics modeling, symbolic knowledge infusion, AI in clinical decision support

Author “Wei Li” was erroneously assigned as corresponding author. The correct corresponding author is “Shu Chen”.

The original version of this article has been updated.

